# *In Vitro* Activity of Carbapenems Alone and in Combination With Amikacin Against KPC-Producing *Klebsiella Pneumoniae*

**DOI:** 10.4021/jocmr551w

**Published:** 2011-05-19

**Authors:** Jennifer Le, Barbara McKee, Warunee Srisupha-Olarn, David S. Burgess

**Affiliations:** a University of California, San Diego, Skaggs School of Pharmacy and Pharmaceutical Sciences, 9500 Gilman Drive, MC 0714, La Jolla, CA 92093-0714, USA; b Long Beach Memorial Medical Center, 2801 Atlantic Ave, PO Box 1428, Long Beach, CA 90801-1428, USA; c Center for Advancement of Research and Education in Infectious Diseases, University of Texas, College of Pharmacy, Austin, TX, USA; d Pharmacotherapy Education and Research Center, School of Medicine, The University of Texas Health Science Center San Antonio, 7703 Floyd Curl Drive- MSC 6220, San Antonio, Texas 78229-3900, USA

## Abstract

**Background:**

Although carbapenems are the primary treatment strategy for invasive infections caused by ESBL bacteria, case reports of these pathogens with reduced carbapenem susceptibility have emerged. One potential treatment modality is to optimize the use of anti-infectives with combination therapy. We evaluated the activity of carbapenems alone and in combination with amikacin against these clinical isolates.

**Methods:**

Time-kill studies evaluated ertapenem (ETP), imipenem (IPM), meropenem (MEM), and amikacin (AMK) against 4 non-duplicate clinical isolates of *Klebsiella pneumoniae* that were resistant to these antibiotics. Synergy was defined as ≥ 2 log_10_ decrease CFU/mL at 24 h for the combination when compared with the most active single agent of the combination, plus the number of surviving organisms for the antimicrobial combination was ≥ 2 log_10_ less than the initial inoculum.

**Results:**

All isolates carried *bla_KPC-3_* and genes encoding TEM-1 and SHV-11/-36; and were resistant to carbapenems (MIC at ≥ 8 μg/mL for ETP, MEM and IPM) and AMK (MIC 32 μg/mL) using broth microdilution. As monotherapy, none of the carbapenems nor AMK achieved and maintained bactericidal activity defined as ≥ 99.9% or > 3 log_10_ killing. From time-kill studies, synergy was demonstrated for MEM and IPM in combination with AMK over the entire 24 h against all isolates. In addition, MEM and IPM with AMK achieved and maintained bactericidal activity (≥ 99.9% killing) at 24 h against 2 and 1 isolate(s), respectively. Bactericidal activity and synergy were not observed for ETP combinations.

**Conclusions:**

The combination of MEM or IPM with AMK displayed synergistic activity against KPC-3-producing *K. pneumoniae* isolates.

**Keywords:**

ESBL; *Klebsiella pneumoniae*; KPC; Carbapenemase; Time-kill; Meropenem; Amikacin; Imipenem; Ertapenem; Carbapenem; Synergy

## Introduction

Carbapenems are the primary treatment strategy for serious infections caused by extended-spectrum beta-lactamase (ESBL)-producing *Klebsiella pneumoniae*. However, production of KPC β-lactamase, a serine carbapenemase primarily detected in *K. pneumoniae*, has emerged in the USA and is probably contributing to carbapenem resistance rates among Enterobacteriaceae [1, 2].

In light of this carbapenem resistance and with the dearth of new antimicrobial agents with novel mechanisms of action, combination therapy using existing antibiotics becomes an attractive option. The combination of two antibiotics from different classes will increase the probability that at least one antibiotic will provide adequate activity against the multi-drug resistance isolate and enhance their activity when synergy is present. This is crucial since inappropriate initial antimicrobial therapy has been associated with increased mortality [3-5].

Based on limited clinical experience, susceptibility data and single antibiotic pharmacokinetic-pharmacodynamic studies, polymixin B [6, 7], tigecycline [6-9], AMK [6], carbapenems [10-12], and clavulanic acid like ticarcillin/clavulanate [13] may play a role in combination therapy for infections caused by KPC-producing pathogens. We performed time-kill studies to evaluate the *in vitro* activity of carbapenems [including ertapenem (ETP), imipenem (IPM) and meropenem (MEM)] alone or in combination with amikacin (AMK) against KPC-producing *K. pneumoniae*. These antibiotics were selected due to their safety profile and feasibility to be used empirically since detection of KPC production in clinical laboratory can be challenging. In addition, amikacin has a fast rate of bactericidal activity [14] and post-antibiotic effect [15].

## Materials and Methods

Drug stock solutions were prepared according to the manufacturer’s recommendations from powders of ETP (AstraZeneca, Wilmington, DE), IPM (Merck, Whitehouse Station, NJ), MEM (AstraZeneca, Wilmington, DE), and AMK (Bristol-Myers Squibb, New York, NY). Drug concentrations were based on the mean steady-state serum concentration obtained using normal doses of each antibiotic in normal healthy volunteers. The following antibiotics at the specified concentrations (g/mL) were used: ETP 2, IPM 4, MEM 4, and AMK 16.

Four non-duplicate isolates of *K. pneumoniae* obtained from an observational clinical outcomes study carried *bla_KPC-3_*italic and genes encoding TEM-1 and SHV-11/-36 [16]. Isolate number 16, 17, 38 and 50 corresponded to the following patients 1, 2, 4 and 3, respectively. Isolates were negative for all other beta-lactamase encoding genes, including plasmidic AmpC, serine- and metallo-β-lactamases, tested. Molecular typing demonstrated that all 4 KPC-3-producing strains were clonally related.

Using broth microdilution as described by the Clinical and Laboratory Standards Institute (CLSI), all strains were resistant to carbapenems (MIC at ≥ 8 μg/mL for ETP, MEM and IPM) and AMK (MIC 32 μg/mL). Likewise using E-tests, MIC values of all carbapenems were ≥ 32 μg/mL for all isolates and AMK were 128, 32, 48 and 48 μg/mL for C16, C17, C38 and C50, respectively.

Time-kill studies were performed using a final volume of 25 mL of cation-adjusted Mueller-Hinton broth and an initial inoculum of ~ 1 x 10^6^ CFU/mL. Samples were taken at 0, 4, 8, 12 and 24 h. Fifty μL was plated on trypticase soy agar plates using a spiral plater (Spiral Biotech, Bethesda, MD) and then incubated at 35 ^o^C for 24 h before colony counts were determined using a laser colony counter (CASBA 4, Spiral Biotech, Bethesda, MD). The spiral plater controlled antibiotic carry-over [17]. Time-kill profiles were constructed (log CFU/mL versus time). The limit of quantification was 10^2^ CFU/mL. Any colony count lower than this limit was rounded to 10^2^ CFU/mL. All tests were performed in duplicate.

## Results

**Table 1:** Change in Colony Count (log_10_10 CFU/mL) at 24 Hours Compared to Initial Inoculums for KPC-3-Producing *K. Pneumoniae* Isolates Using Time-Kill Methodology

Antibiotic	C16	C17	C38	C50
Growth control	5.05	4.13	4.78	4.55
AMK	1.36	3.75	2.98	4.23
ETP	4.00	3.14	5.16	4.83
IPM	4.02	3.30	4.50	4.71
MEM	3.69	3.59	4.90	4.09
ETP + AMK	1.57	2.81	3.86	4.45
IPM + AMK	-1.48*	-0.05*	-2.13*	-3.53*
MEM + AMK	-1.71*	-2.61*	-3.58*	-3.07*

Synergy denoted as *. AMK: amikacin; ETP: ertapenem; IPM: imipenem; MEM: meropenem.

**Figure 1.**
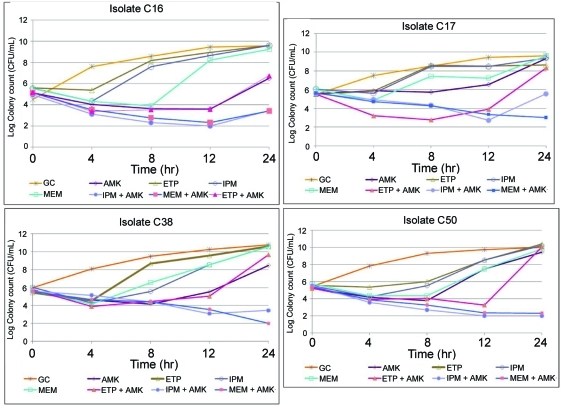
Time-kill studies of carbapenems and amikacin alone and in combination against four KPC-3-producing *K. pneumoniae* isolates. GC: Growth control; AMK: amikacin; ETP: ertapenem; IPM: imipenem; MEM: meropenem.

The mean starting inoculum for all of the time-kill experiments was 5.51 × 10^6^ CFU/mL with an intra-day CV of < 7.0%. As monotherapy, none of the carbapenems nor AMK achieved and maintained bactericidal activity defined as ≥ 99.9% or > 3 log_10_ killing (Table 1). Maximum killing of ≥ 90% was observed for MEM and AMK against all isolates; and IPM against 3 isolates (except C17). ETP alone was least active as it did not achieve ≥ 90% killing in any isolates. Antibacterial activity was not maintained since significant regrowth was observed for all antibiotics after achieving 90% killing against each of the isolates (Fig. 1). In particular, amikacin demonstrated regrowth after 8 hrs.

Time-kill curve results for each carbapenem in combination with AMK are depicted in Figure 1. Synergy was defined as ≥ 2 log_10_ decrease CFU/mL at 24 h for the combination when compared with the most active single agent of the combination, plus the number of surviving organisms for the antimicrobial combination was ≥ 2 log_10_ less than the initial inoculum. Synergy was demonstrated for MEM and IPM in combination with AMK over the entire 24 h against all isolates. In addition, MEM and IPM with AMK achieved and maintained bactericidal activity (≥ 99.9% killing) at 24 h against 2 isolates (C38 and C50) and 1 (C50), respectively. For these isolates C38 and C50, the time to bactericidal activity were 12 h for MEM plus AMK and 24 and 8 h, respectively, for IPM plus AMK.

Bactericidal activity and synergy were not observed for ETP combinations. In addition, significant regrowth for ETP with AMK was evident for all isolates between 8 and 12 h. Slight regrowth occurred in 1 isolate (C16) for MEM and 3 isolates (except C50) for IPM combinations at 24 h. None of the antibiotic combinations demonstrated antagonism, which was defined as ≥ 2 log_10_ increase in colony count at 24 h for the combination when compared with the most active single agent of the combination.

## Discussion

The clinical utility of carbapenem monotherapy is limited against isolates that produce KPC carbapenemases [13]. Studies of prolonged infusion of high-dose regimens of beta-lactams, particularly meropenem at 2 g every 8 hours over 3-hours, against KPC isolates have produced conflicting results [18, 19]. Although rapid colony reduction was initially observed, regrowth occurred after 6 hr of meropenem exposure. Furthermore, meropenem exposure (or the percent time of free drug concentration above the MIC) for KPC isolates with elevated MICs of 8 and 16 μg/mL was significantly reduced due to rapid *in vitro* hydrolysis [19].

In light of the emerging resistance to carbapenems and the limited utility of overcoming this resistance through prolonged infusion of high-dose carbapenem monotherapy, combination therapy may play a role in the treatment of infections associated with KPC production since it may provide the potential for synergistic effects between two different classes of anti-infectives [20]. Studies evaluating antibiotic combinations against KPC-harboring isolates are limited. In our study, MEM or IPM in combination with AMK displayed synergy against KPC-producing *K. pneumoniae*. In another study, synergy was observed in combinations of polymyxin B with rifampin, doxycycline or tigecycline [7, 21].

### Conclusion

The addition of AMK to carbapenems, particularly MEM and IPM, provided synergistic effects and enhanced bactericidal activity against *K. pneumoniae* with reduced susceptibility to carbapenems. Prolonged infusion of a high-dose carbapenem in combination with other potentially active antibiotics (such as AMK, tigecycline, and polymyxin B) may be a therapeutic option for serious infections associated with KPC production. Further investigation is warranted to determine if our *in vitro* observations translate into clinical efficacy. In addition, more research is imperative to optimize the dosing regimens of currently approved antibiotics using pharmacokinetic-pharmacodynamic principles and to explore the potential role of novel chemical entities.
